# Culturally centering alcohol use assessments: insight from indigenous adults with lived and living experience

**DOI:** 10.3389/fpubh.2026.1834637

**Published:** 2026-06-17

**Authors:** Katherine A. Hirchak, Kelsey Bajet, Kellie Webb, Sharon Wagon, Ray Daw, J. Scott Tonigan, Melissa Walls, Kamilla L. Venner

**Affiliations:** 1 Department of Community and Behavioral Health, Elson S. Floyd College of Medicine, Washington State University, Spokane, WA, United States; 2 Promoting Research Initiatives in Substance Use and Mental Health Collaborative, Washington State University, Spokane, WA, United States; 3 Connections in Indigenous Research, Cultural Leadership, Equity, & Solidarity, Washington State University, Spokane, WA, United States; 4 Tribal Healing Center, Wyoming, WY, United States; 5 Behavioral Health Consultant, Albuquerque, NM, United States; 6 Department of Psychology and Center on Alcohol, Substance use, And Addictions (CASAA), University of New Mexico, Albuquerque, NM, United States; 7 Center for Indigenous Health, Johns Hopkins University, Baltimore, MD, United States

**Keywords:** alcohol assessments, American Indian and Alaska Native communities, community engaged, culturally adapted measures, qualitative

## Abstract

**Objectives:**

There are high rates of both alcohol abstinence and hazardous drinking among American Indian and Alaska Native (AI/AN) communities compared with non-Hispanic Whites. Few measures exist that examine alcohol use consequences within a relevant historical and cultural context for Indigenous populations, hindering the ability of communities, researchers, and practitioners to effectively address alcohol misuse. In the present study, we discuss a research collaboration to culturally re-center alcohol-use consequence measures among AI/AN adults.

**Methods:**

We convened a national and local Scientific Review Panel (SRP) with AI/AN research and clinical experts to reexamine the Drinker Inventory of Consequences (DrInC) and the companion measure, the Short Inventory of Problems (SIP; *N* = 5). We then completed 20 interviews with AI/AN adults currently in alcohol recovery or misusing alcohol. Interview questions included the alignment of items within measures to be more culturally relevant, identifying missing content, and a review of the research team’s adaptations. We completed a thematic analysis.

**Results:**

The SRP focused their adaptations on re-centering culture, assessing risk and protective factors, and important missing item content elements. Ultimately, the SRP recommended removing 20 items from the DrInC and adding 12 items to the SIP and adding 3 sub-scales, with an emphasis on the addition of items that were strengths-based and removal of stigmatizing language. Interviews were then completed to further refine the measures. In total, three themes emerged from these data: (1) Direct Reflections on Original and Adapted Items; (2) Negative Alcohol Consequences Specific to AI/AN Communities; and (3) Recovery Supports.

**Conclusion:**

Findings underscored the need to emphasize culture, prevention, and recovery supports when addressing alcohol misuse with AI/AN adults. The SRP also identified areas to expand the culturally centered framing of these measures by removing stigmatizing language, focusing on systemic factors, and prioritizing strengths-based approaches. The subsequent culturally centered tools will be validated with 150 nationally recruited AI/AN adults.

## Introduction

There are many cultural factors in American Indian and Alaska Native (AI/AN) communities that support individual wellbeing and recovery from alcohol and other substance use. Some of these factors include land-based healing, service to Elders, extended families and community networks, drumming, and spirituality (1, 2). In the United States (US), AI/AN adults have higher prevalence rates of past-month alcohol *abstinence* compared to non-Hispanic Whites (NHW; 58–60% vs. 43%, respectively) (3). However, AI/AN adults also report higher rates of severe alcohol use disorders compared with other ethnocultural groups (4). Thus, despite high rates of non-drinking, public health consequences of alcohol misuse may be elevated within some Indigenous communities.

In addition to political and historic components, such as policies resulting in genocide, land theft, boarding schools, and spiritual persecution, that contribute to health disparities and inequities related to alcohol dependence (5), AI/AN adults are also more likely than non-AI/AN adults to have co-morbid substance use and psychiatric symptoms (6). Potentially resulting in differential impacts of alcohol use and accompanying negative drinking consequences (7, 8). Therefore, assessing drinking consequences is an important component for clinicians and researchers seeking to improve treatment with AI/AN communities.

### Culturally centering measures with AI/AN adults

A limited number of alcohol use measures have been developed with AI/AN people (9). These limited measures might be categorized into two groups: culturally centered (e.g., existing measures adapted to meet community needs) (10) and culturally grounded (e.g., measures developed within the community) (11). Among the surveys that do exist is a widely used assessment called the Addiction Severity Index-Native American Version (ASI-NAV) (12). The ASI-NAV is an 8-domain alcohol use survey that has been normed and adapted among AI/AN adults. Limitations of adapted measures, such as this, include communities working to fit questions into their clinical contexts that may not be relevant to them. Alternatively, culturally grounded measures that include holistic components have focused on social support and cultural strengths to address suicide and alcohol overuse, primarily among youth (13–15). While culturally grounded measures are critical to increase validity, barriers for communities to pursue the development of such measures include high costs and time burden. Therefore, instead of simply using an unadapted measure and retrospectively assessing psychometric properties (13), culturally centering existing measures provide the added utility of integrating culturally appropriate constructs into an instrument (e.g., relationality and connection and cultural activities) (16) that can be rapidly integrated into clinical practice. Here, we describe our efforts to culturally center two existing measures for negative alcohol use consequences.

### A brief overview of the drinker inventory of consequences (DrInC) and the short inventory of problems (SIP)

The DrInC was developed in the 1990s as a self-report questionnaire to comprehensively assess alcohol-related problems distinct from alcohol consumption. The measure consists of 50 items, and a shorter version, the SIP, was condensed to 15-items to assist with rapid assessment in busy clinical practice settings (17). The DrInC assesses five domains related to drinking consequences: (1) physical, (2) intrapersonal, (3) social responsibility, (4) interpersonal, and (5) impulse control. Sample items include, “My drinking has gotten in the way of my growth as a person” and “My drinking has damaged my social life, popularity, or reputation.”

Efforts were made in the item development of the DrInC to include drinking consequences that might impact women more significantly (e.g., effects on parenting), but the measure was normed on a primarily non-Hispanic White (NHW) sample (i.e., 84% of participants were NHW) (17). Although not developed with a diverse sample, the DrInC has since demonstrated good psychometric properties among diverse populations, including adults with serious mental illness and sexual and gender minorities (18–20). Most relevant to the current study, previous research indicated the preliminary value of using the SIP in assessing the negative impact of drinking among urban AI/AN adults (16). Results suggested that drinking differentially impacts male and female AI/AN adults. Women experienced more negative consequences related to increased alcohol consumption, whereas men who had higher abstinence demonstrated fewer negative consequences across all five SIP domains (16). Therefore, further gender-based and culturally centered measurement may be necessary to more fully capture the range of negative drinking consequences among AI/AN adults (16). By examining the impact of negative drinking consequences, individuals may increase their awareness of problems with alcohol, while clinicians can identify characteristics associated with less favorable outcomes (21, 22). Furthermore, identification of alcohol problems can potentially lead to motivation to change, which has also been related to improved treatment outcomes (21, 23, 24).

### Present study

In the present study, we describe our research collaboration to refine alcohol use consequence assessments with AI/AN adults. We convened a Scientific Review Panel (SRP) that guided all aspects of the research. Our focus was to culturally center the DrInC and SIP measurement tools to capture constructs important to alcohol consequences and recovery among AI/AN communities that may not have been included in the original version. Qualitative interviews were subsequently completed with 20 AI/AN adults. Results add to the limited literature on improving alcohol-related measures with Indigenous populations.

## Methods

### Tribal sovereignty

All research was approved by the two respective Tribal Business Councils, which include data sharing agreements and accompanying Tribal Resolutions. Community representatives were also included on a national board that oversees the scientific rigor and research ethics of the project. We were also approved under the Rocky Mountain Tribal and Washington State University Institutional Review Boards.

### Positionality and reflexivity

As part of Indigenous-centered praxis in qualitative research, positionality statements have been included for authors. We include the information to note how we have come to the research and that our subjective experiences and perspectives shape us as researchers (25). KH is a descendant of the Eastern Shoshone Tribe and has mainly Italian European ancestry. For more than 15 years, she has partnered with AI/AN communities. KB is Filipino and a descendant of immigrants. For more than 4 years, she has worked with AI/AN communities. KW is Cowlitz and Eastern Shoshone. She has 20 + years of clinical and research experience. SW is Eastern Shoshone with 20 + years of experience serving her community. RD is Diné and has more than 30 years of experience as a clinician supporting the health and wellbeing of Indigenous communities. JST is non-Indigenous, assisted in the original development of the DrInC and SIP, and has collaborated on research with AI/AN communities for more than 20-years. MW is Eagle Clan and a first-generation descendant of the Couchiching First Nation and Bois Forte Band of Ojibwe and of Swedish/German descent. She has over two decades of experience in research partnerships with Indigenous communities. KV is an Athabascan Tribal member with 30 + years of experience in supporting the health and wellbeing of AI/AN communities.

### Original and adapted DrInC and SIP measures

The original DrInC and SIP, consisting of 50 items and 15 items, respectively, assess five domains: physical, intrapersonal, social responsibility, interpersonal, and impulse control. The DrInC for Alaska Native (DrInC-AN) was adapted by Allen (26) consists of seven domains: physical, intrapersonal, social responsibility, interpersonal, impulse control, spirituality, and kinship loss (26). Many of the items from the original DrInC were pulled into the new measure with slight adaptations that were more relevant to Alaska Native adults. For example, “Have you driven a boat, car, or snowmachine after drinking?” Other items were switched around across domains, and new items were added to create the more culturally relevant domains of spirituality and kinship loss.

Items under the interpersonal domain were switched with items from the social responsibility domain in the DrInC-AN. For example, the DrInC interpersonal item, “My family has been hurt by my drinking,” was moved under the social responsibility domain in the DrInC-AN and reworded to “Have your relatives been hurt by your drinking?” The DrInC social responsibility item, “I have failed to do what is expected of me because of my drinking,” was moved under the interpersonal domain in the DrInC-AN and reworded to “Have you failed to do what others expect of you because of your drinking?” Language changes were made to reflect local expressions. For example, “When drinking, I have done impulsive things that I regretted later” was changed to “When drinking, have you done things without thinking, and wished you had not done them later?”

### Adaptation framework and scientific review panel-national and local

We completed our research in the spirit of Community-Based Participatory Research (CBPR). CBPR is a best-practice framework for Tribal communities that is collaborative and strengths-based (27). CBPR has led to improved health and social equity outcomes among Indigenous communities (28, 29). We loosely employed the Culturally Specific Prevention framework in our adaptation process. The framework includes five stages that we applied broadly: (1) researchers identify risk and protective factors; (2) these factors are then examined among the AI/AN community; (3) cultural experts translate the factors into the specific context which culminates in (4) the development of measures of risk and protective factors that fit the cultural context and (5) trials of the culturally specific intervention are subsequently conducted (30, 31). We focused on the first four stages to guide the initial cultural centering of these measures.

We convened a Scientific Review Panel (SRP) that included AI/AN academic researchers, AI/AN community researchers, clinicians, and experts in measurement development and treatment research (*N* = 5). A member of the SRP was also a site PI for the rural reservation community partner, and another was an Elder. The primary feedback from the SRP was to align the measure(s) to focus on resilience and update language around alcohol use. For a better cultural fit, the SRP suggested a strengths-based approach to move away from a deficit model. The Medicine Wheel was recommended to conceptualize the emotional, physical, mental, and spiritual domains of health and recovery. One member suggested adding protective factors, strengths, and resiliency as construct content areas.

For reframing the items, emphasis was on language and the importance of not shaming participant-relatives and removing potentially stigmatizing language. The SRP also recommended wording and adaptations that centered on the person struggling with alcohol misuse. It was also advised that cultural adaptations should be considered if items affect how participant-relatives view themselves. Items also needed to include the impacts of alcohol use on families and communities. Suggested edits should emphasize behaviors and cultural engagement. In addition, the SRP was enlisted in several research activities, including the development of the interview questions, making adaptations to the original items, developing a codebook, and interpreting the findings.

### Participant-relatives and recruitment

Using purposive sampling, participant-relatives (i.e., culturally preferred term that underscores relationality and interconnectedness of study participants and the community) were recruited for qualitative interviews through a partnership with a Healing Center located on a rural reservation. Flyers were distributed by local reservation service providers, program websites, and social media pages within the community (e.g., a service program’s Facebook). National recruitment was completed through a company that assists with the recruitment of people in recovery and treatment spaces. Recruitment through this source included an online platform operated by the company that invited urban AI/AN adults from across the U.S. to participate. While the recruitment efforts included a rural reservation partnership, most of the participant-relatives were recruited nationally.

Eligibility criteria was entirely based on self-report and included: (1) willing and able to provide informed consent; (2) self-identifying as an AI/AN adult (18 + years old); (3) internet access and device for messaging/contact; (4) fluent in English; (5) using alcohol or in recovery; (6) or a family member of someone currently using substances or currently in recovery. Exclusion criteria included: (1) traumatic brain injuries or other history of seizures; and (2) psychiatric or cognitive conditions that preclude providing informed consent. No one declined to participate after study procedures were described. Since all inclusion criteria were based on self-report, we could not independently verify the accuracy of what was reported.

Qualitative interviews were completed using cognitive interviewing, a technique where participant-relatives think out loud and paraphrase items to understand their thinking and comprehension. We included several judgment (e.g., knowledge, attitude about the topic), retrieval (e.g., relevant), and understanding (e.g., wording clear and easy to understand) questions to understand experience and comprehension (32). Interviews lasted about 60 min and were conducted via phone or Zoom and recorded for audio transcription. Participant-relatives were compensated $25 for their participation.

### Interview question development

A Semi-Structured Interview Guide was developed prior to the interviews under the guidance of the panel. The interview questions focused on the lived/living experiences of those who have been negatively impacted by alcohol use, in addition to how the measure might best capture the impact of alcohol misuse on participant-relatives’ lives and on their culture. For example, a question focused on appropriateness of the wording of measure questions included “Do you think any of the questions make you experience shame/stigmatize participants and the community?” When asking about the negative impacts of alcohol use, “Does alcohol misuse negatively impact Native cultures/you/your family? If so, what are examples?” Strengths-based questions included, “Are there traditional activities or cultural activities that you like to do that stop you from using alcohol?” We pilot tested the questions with early-stage AI/AN researchers and research coordinators to improve clarity, timing, and repetition of the questions.

### Data analysis

An exploratory hybrid inductive and deductive coding process was used. A deductive approach was used to assess when responses related to culture or substance-free activities fell within the domains of the medicine wheel: emotional, physical, mental, and spiritual. Two members of the research team independently reviewed 25% of the interview transcripts that were selected at random. Codes were then discussed, with codes consolidated and decided upon by consensus between the two coders. The codebook draft was sent to the panel for review and feedback, with a request for naming any potential missing content areas or codes. A total of 44 codes were ultimately derived from these data, such as motivation to change and recovery tools. Once feedback had been provided and integrated into a finalized codebook, the two coders coded all transcripts in Dedoose. The lead author then completed a content analysis of the excerpts using a qualitative description approach, analyzing all the coded data by identifying key themes that emerged and exemplar quotes that encompassed experiences across the interviews. Interpretation of these data were discussed with the community-based staff and clinicians for internal face validity. Members of the Scientific Review Panel also provided high-level reflections on data interpretation.

## Results

### SRP feedback

**Figure 1:**
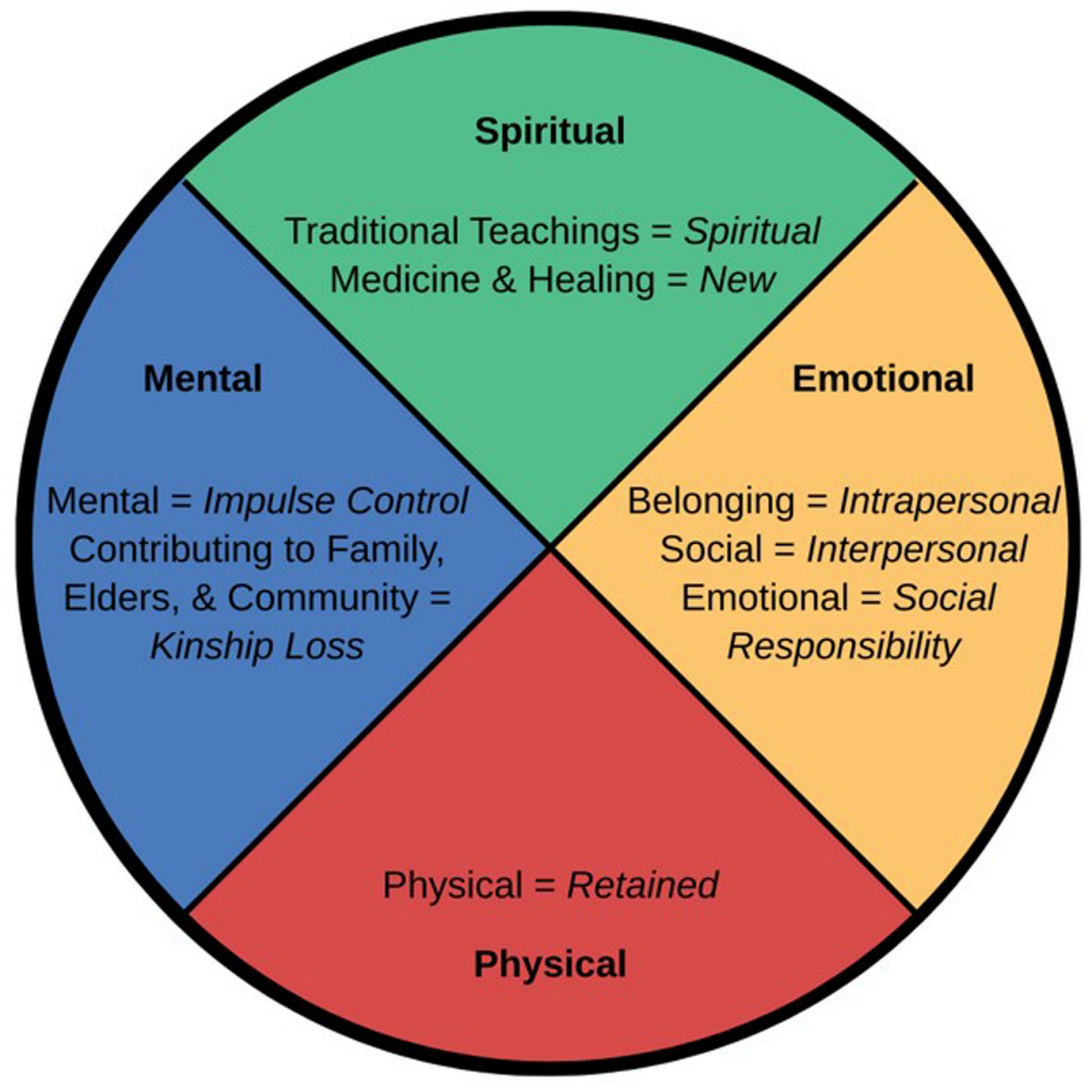
Recentered subscales: the medicine wheel.

The domains for the adaptation were suggested to reflect the Medicine Wheel. The Medicine Wheel is a holistic approach to health that considers the emotional, physical, mental, and spiritual domains of health (Figure 1). The SRP advised that the content of the items for *Intrapersonal* aligned with many AI/AN communities’ beliefs related to the *Emotional* domain and renamed the sub-scale *Belonging* (Table 1). *Interpersonal* was also reconceptualized, and it was agreed that it fell within the values of *Emotional*, but with a greater emphasis on the social components. It was therefore renamed *Social*. *Social Responsibility* was broadly conceptualized as *Emotional*. *Physical* was retained as is, given that the sub-scale already encompassed the behaviors or outcomes associated with alcohol use (Table 2). *Impulse Control* was expanded and revised to *Mental*, as the item content contained opportunities for reflection, learning, and decision-making (Table 3).

**Table 1:** Recentered measures: emotional.

	DrInC-original	Adapted	
Q#	Intrapersonal (sub-scale)	Belonging (adapted sub-scale)	Q#
2	I have felt bad about myself because of my drinking		
12	I have been unhappy because of my drinking	Have you felt depressed, sad, unhappy or isolated because of your alcohol use?	8
16	I have felt guilty or ashamed because of my drinking	Have you felt guilty or ashamed because of your alcohol use?	6
18	When drinking, my personality has changed for the worse	While using alcohol, has your personality changed for the worse?	7
34	I have lost interest in activities and hobbies because of my drinking	Have you lost interest in activities and hobbies because of your alcohol use?	5
36	My spiritual or moral life has been harmed by my drinking		
37	Because of my drinking, I have not had the kind of life that I want	Has your life changed in a negative way (for example, DUI, jail) because of your alcohol use?	9
38	My drinking has gotten in the way of my growth as a person	Has your alcohol use gotten in the way of your growth as a person?	10
Q#	Interpersonal (sub-scale)	Social (adapted sub-scale)	Q#
4	My family or friends have worried or complained about my drinking	Have you felt bad about yourself because of your alcohol use?	11
7	My ability to be a good parent has been harmed by my drinking	Have you failed to follow through on relationship expectations because of your alcohol use (for example, family, community, friendships, other kinship relationships)?	12
17	While drinking, I have said or done embarrassing things	Have you had money problems, or been short of food or clothes, because of alcohol use?	13
21	While drinking or using drugs, I have said harsh or cruel things to someone		
27	My marriage or love relationship has been harmed by my drinking		
30	My family has been hurt by my drinking		
31	A friendship or close relationship has been damaged by my drinking		
39	My drinking has damaged my social life, popularity, or reputation		
43	I have lost a marriage or a close love relationship because of my drinking		
46	I have lost a friend because of my drinking		
Q#	Social responsibility (sub-scale)	Emotional (adapted sub-scale)	Q#
3	I have missed days of work or school because of my drinking	Has your alcohol use hurt your social life or reputation?	15
6	The quality of my work has suffered because of my drinking	Have you lost friends or your social group due to your alcohol use?	17
14	I have failed to do what is expected of me because of my drinking	Have you failed to do what is expected of you by your family or community because of your alcohol use?	14
20	I have gotten into trouble because of drinking	Have people stopped talking to you because of your alcohol use?	18
26	I have had money problems because of my drinking		
40	I have spent too much or lost a lot of money because of my drinking		
44	I have been suspended/fired from or left a job or school because of my drinking	Have you been suspended, fired from, or left a job/school because of alcohol use?	16

**Table 2:** Recentered measures: physical.

	DrInC	Adapted	
Q#	Physical (sub-scale)	Physical (adapted sub-scale)	Q#
1	I have had a hangover or felt bad after drinking	Have you had a hangover after alcohol use?	3
8	After drinking, I have had trouble sleeping, staying asleep, or nightmares	After using alcohol, have you had trouble with your sleep (e.g., sleep patterns, falling asleep, staying asleep, or having nightmares?)	1
11	I have been sick and vomited after drinking		
13	Because of my drinking, I have not eaten properly		
24	My physical health has been harmed by my drinking		
33	My sex life has suffered because of my drinking		
29	My physical appearance has been harmed by my drinking	Has your physical health/appearance/hygiene negatively changed because of your alcohol use?	4
48	While drinking or intoxicated, I have been physically hurt, injured, or burned	While using alcohol or under the influence of alcohol, have you ever experienced unintentional injury?	2

**Table 3:** Recentered measures: mental.

	DrInC	Adapted	
Q#	Impulse control (sub-scale)	Mental (adapted sub-scale)	Q#
9	I have driven a motor vehicle after having three or more drinks		
10	My drinking has caused me to use other drugs more		
19	I have taken foolish risks when I have been drinking	Have you gotten into a physical fight or done foolish or dangerous things after using too much alcohol?	20
22	When drinking, I have done impulsive things that I regretted later	When using alcohol, have you done things without thinking, and wished you had not done them afterwards?	19
23	I have gotten into a physical fight while drinking	Have you gotten into a physical fight or done foolish or dangerous things after using too much alcohol?	20
28	I have smoked tobacco more when I am drinking		
32	I have been overweight because of my drinking		
41	I have been arrested for driving under the influence of alcohol		
42	I have had trouble with the law (other than driving while intoxicated) because of my drinking		
47	I have had an accident while drinking or intoxicated		
49	While drinking or intoxicated, I have injured someone else	While using alcohol, have you hurt someone else?	21
50	I have broken things while drinking or intoxicated		
Q#	Kinship loss (DrInC-AN)	Contributing to family, elders, and community (adapted sub-scale)	Q#
		Has your alcohol use hurt your ties with a friend or relative?	25
		Do you feel like your ability to be a good parent has been harmed because of your alcohol use?	26
		Do you feel like your alcohol use damaged your ability to be a good community member, or your reputation or your role in your community?	27
		Have you lost custody of a child because of your alcohol use?	28

In addition, the SRP preferred the Alaska Native version that included a new subscale of *Kinship Loss*. The SRP also recommended a focus on the impacts of alcohol misuse on the broader community and social support. *Kinship Loss* was subsequently renamed *Contributing to Family, Elders, and Community* to be inclusive of these additional dimensions. The Alaska Native version also included a *Spiritual* domain. In addition to *Spiritual*, the SRP described the importance of cultural activities, so they added a sub-scale of *Traditional Teachings* (Table 4). Finally, they emphasized the need for more prevention and strengths-based approaches to wellbeing, so they added an entirely new sub-scale of *Medicine & Healing*. Ultimately, the SRP recommended removing 20 items from the DrInC and adding 12 items to the SIP. Eight total sub-scales were included, expanded from the original 5: two adapted sub-scales from the Alaska Native version and one new sub-scale (Table 4).

**Table 4:** Recentered measures: spiritual.

	DrInC-AN	Adapted	
Q#	Spiritual (DrInC-AN)	Traditional teachings	Q#
		Have you not gone to traditional ceremonies, church, spiritual gatherings, giveaways, round dances, or feasts because of your alcohol use?	22
		Have you not felt spiritually at peace because of your alcohol use?	23
		Has your engagement in spirituality/prayer been hurt by your alcohol use?	24
Q#	New subscale	Medicine and healing	Q#
		Do you feel like going to sweats stops you from using alcohol? (SIP)	29
		Do you feel like participating in a ceremony/or using plant medicines (e.g., cedar, sweet grass, sage) stops you from using alcohol? (SIP)	30
		Do you feel like beading or other cultural activities stop you from using alcohol? (SIP)	31
		Do you feel like exercising stops you from using alcohol?	32
		Do you feel like playing your favorite sport stops you from using alcohol?	33
		Do you feel like AA/peer support/family stops you from using alcohol?	34
		Do you feel like spending time with your family and friends stops you from using alcohol?	35
		Do you feel like horse culture stops you from using alcohol?	36
		Do you feel like your favorite hobby/activity stops you from using alcohol?	37
		Do you feel like singing or dancing stops you from using alcohol?	38
		Do you feel like talking to an Elder or having an Elder that you can talk to stops you from using alcohol?	39
		Do you feel like being employed/being enrolled in school stops you from using alcohol?	40

### Participant-relative demographic characteristics

In total, 20 semi-structured qualitative interviews were completed with AI/AN adults. Participant-relatives were mostly women (70%) and on average 41.3 years old. Approximately 50% of relatives had an Associate’s or Bachelor’s Degree. Most relatives lived in urban locations (90%), and 85% were stably housed, and 45% of relatives had a full-time job (Table 5).

**Table 5:** Sample characteristics.

Demographics	Participants-relatives
	*N* = 20
Age	41.3 (14.3)
Sex	
Female	70%
Male	25%
Two-spirit	5%
Education	
Less than High School	5%
High School Diploma or Equivalent	25%
Some College/Associate’s Degree	30%
Bachelor’s Degree or Higher	40%
Employment	
Unemployed	10%
Student	15%
Part-time	15%
Full-time	45%
Retired/disability	15%
Housing	
Stably housed	85%
Temporary housing/houseless	15%
Location	
Urban	90%
Rural	10%

Percentages may not equal 100% due to rounding.

### Theme 1. Direct reflections on original and adapted items

Participant-relatives provided a range of feedback. The views expressed across interviews primarily related to: (1) item content, (2) question format, (3) measure administration, and (4) cultural adaptations. Participant-relatives commented that none of the questions, either in the original or adapted versions, felt inappropriate or not culturally responsive. Only one person commented that the original items might not feel as accessible, describing them as “white.” Participant-relatives commented that the original DrInC had an individual focus and was not specific enough to an individual’s environment. While there were varied opinions, generally participant-relatives preferred the holistic adapted versions. They also commented on colonial/social determinants of health, such as the density of alcohol outlets in certain neighborhoods. One participant-relative commented,

The first original survey, it’s like you said it did not… it seemed very broad— I’m trying to think of how to put it. It did not take into cultural or economic, maybe, situations that may be affecting people’s lives as to why they consume alcohol or the amount of alcohol they consume and availability and—Like, in rich white neighborhoods, there’s not a liquor store on every corner. …kind of, broad general questions rather than—to me, they could have been more specific and had fewer questions, and it would have gotten the same job done.

– 2034 (Female, 46 years old).

Suggestions for content additions were related to justice involvement, income, formal treatment history, and a stronger focus on holistic wellness (e.g., exercise, nutrition, and specific cultural activities). Some participant-relatives observed that certain questions were repetitive, but also thought it appropriate and reasonable to encompass all the negative consequences. In addition, participant-relatives liked the simplicity of the binary format of the items. They were mixed on preference for interview style “you” versus “I” statements in the questions.

There were also suggestions related to administering the measures. People expressed mixed views on whether they preferred a longer or shorter measure, but most did not object to the longer version. For example, one suggestion was that the shorter questionnaire be administered when people were in the early stages of recovery and might not be feeling well or are unable to answer questions accurately, while the longer measures could be completed with those in mid- or more stable recovery. A few participant-relatives thought people should fill out the measures on their own, while others thought it would be a good tool for clinicians to administer to help them think about how to better meet their clients’ needs. Ultimately, participant-relatives believed that preferences would vary, so clinicians or those administering the measure should not assume one way or another, but could ask.

One positive aspect of the measures noted by participant-relatives was the opportunity to enhance self-awareness and identify areas for change. Relatedly, no one thought the measures themselves were stigmatizing or inappropriate, but many participant-relatives discussed shame around their alcohol use history. The nuanced difference is captured in the following personal reflection, where one person noted shame, but also how the measures would be good for individual accountability:

I personally feel ashamed. I feel like it breaks down like my daily routine or like things I’ve done throughout my life, far as the drinking and how often I’ve done it, how long I’ve done it, how many situations I’ve put myself into that I should not of. I almost feel like it’s like a timeline of my life or like a journal … So it’s a reminder of things or activities or situations I put myself in the, the past… I mean, is the stigmatized like makin’ people feel bad?… I mean, it might for some, but for me, I feel like it’s more of an eye-opener and more of a reality check. I feel like making me more accountable for my own actions.

−2038 (Female, 48 years-old).

Participant-relatives also noted that an important missing component of the original version of the measures is the cultural and external factors contributing to alcohol misuse (e.g., community or family environment). There was consistency across interviews that the Alaska Native items included more about relationships with others, and the items added by our research team focused on both community and family environment, with participant-relatives preferring the combined approach. Many people spoke positively about the cultural and spiritual questions, while others thought that discussing spirituality was a personal choice and may not be relevant. Certain activities described in the Alaska Native items were flagged as not broadly relatable to Indigenous people (e.g., driving a four-wheeler or snowmachine).

For the items that were developed by the SRP, participant-relatives preferred the shift in focus to holistic health and prevention. However, the particulars of cultural connection or engagement were not uniformly agreed upon. For example, a few participant-relatives noted that cultural dancing as a prevention strategy for alcohol use was not likely, while others felt that it was an important element to prevention and recovery. Participant-relatives mainly highlighted that they preferred the measures that included cultural contexts because they captured a broader range of experiences and were not only focusing on the negative aspects of alcohol use, with several people interviewed noting that not all alcohol use is negative. One person summarized by stating,

“I think that the first version [original DrInC] was really good on focusing on you as a person. …it could help people realize how much they have changed from drinking. The second one [DrInC-AN], I think it focuses more on your relationship with others. And the third one that was adapted by your guys’ research team. I think that really helps someone who drinks focus on everything combined. Like, yourself and other people.”

– 1003 (Female, 18 years old).

### Theme 2. Negative consequences of alcohol misuse specific to AI/AN communities

Negative impacts of alcohol touch upon many factors, from the individual to policy. The interviews centered around: (1) individual factors, (2) interpersonal relationships, family, (3) community, and (4) spirituality. Individual-level impacts included mental health, no longer taking care of yourself (e.g., nutrition, hygiene), depression, and isolation. Physical impacts mainly included hangovers and longer-term issues related to cirrhosis. Participant-relatives also described the colonial/social determinants of health. This was framed as the inability to obtain or retain employment, lack of financial stability, or all finances going towards alcohol. A few people recommended adding questions related to justice involvement captured in the following quote,

Was there…like…"jail time because of your drinking”?…. Okay, well, that could-that could probably go on one of them. [Laughter].

–2028 (Female, 67 years old).

Participant-relatives also described how relationships with family had been lost or eroded due to alcohol, with several people describing losing custody of their children or no longer speaking to family members. Negative consequences were also outlined as loss of contributions to the community, which were perceived as how one might have been in service to their community, whether it be by supporting Elders by chopping wood or through leading a ceremony. One participant-relative said,

I think it limits individual development… it derails people of reaching their potential. …. Damaging to the community in the sense that people who normally be expected to contribute to the development of the good of the Tribe and the community, end up being alcoholic…just limits the growth of the community…

–2003 (Male, 36 years old).

Participant-relatives discussed protocol and social norms around alcohol use during cultural activities. Across participant-relatives, it was agreed that drinking was not appropriate in most cultural settings. However, one participant-relative noted that there were certain activities where people could drink, though it is not culturally or socially acceptable (i.e., powwows). They shared,

When you are participating in your—the cultural activities…dancers, or…if you take part in…feasts…you are not supposed to use alcohol…. If you take-take part…then you do not use alcohol…. But then, there was, like, you know, spectators…people attend that…they are inebriated. …The reputation would be impacted by…family members or friends’ family members…not wanting to…be around them, or…kinda disown them.

–2037 (Male, 40 years old).

Another participant-relative further underscored the incompatibility of alcohol involvement with spirituality and ceremony, stating that drinking has detrimental effects on mental health, family, and the community.

And that affects your whole body because it makes every part of your body sick…it also affects your ability to be social in the community, and you need to be social to plan spiritual…activities and ceremonies and…sweats and stuff. And if you are using, or if a lot of people are using, then all those people are miss-missing out on the benefits of…like, thousands of years of really positive spiritual growth within a people’s—or within the culture.

–2036 (Female, 45 years old).

### Theme 3. Recovery supports

Participant-relatives broadly described what facilitates recovery from alcohol misuse. Conversations described how recovery is supported by: (1) family, community, and social support, (2) substance-free activities, and (3) cultural and spiritual engagement. Community involvement and acts of service supporting Elders were mentioned by many as a form of healing. Social support tended to be broader and not as focused on friends, but included the importance of family and spending time together. A participant-relative said,

I love my family, so us being together kind of helps me. I’ve always been a big family person…. As long as we are together, I do not care what we are doing.

–2025 (Two-Spirit, 29 years-old).

Another important factor in recovery was engagement in cultural activities (e.g., beading, sewing, powwows, sweats, Sundance). Additionally, participant-relatives mentioned that physical activities such as going to the gym, outdoor or land-based activities, and basketball tournaments improved their motivation in recovery. Spirituality was another big component for successful recovery, exemplified by this quote,

…I did have a period of 5 years of recovery previously. And I started doing Fancy Shawl lessons with my cousins and, learning to basketweave and taking language lessons and like helping the Elder’s center and stuff like that. I was really on top of all of that during my period of sobriety. And if I did not have access to that to keep me busy with something that I felt was extremely important and relevant to me, then I probably would not have had that period of sobriety…. I feel like round dances and sweats are a good way to keep people from using alcohol. A round dance is really about coming together as a community and healing one another. It’s just a fun event to bring the community together, and it’s drug and alcohol free. And sweats are a really good way to heal somebody who is struggling, but they have to be sober. And if you are invited to sweat, then, you have to be sober. So, it could increase sobriety if you are invited to a sweat on the regular.

–2039 (Female, 39 years-old).

## Discussion

We completed interviews with 20 AI/AN adults to culturally re-center two widely used alcohol use consequence measures. Our results indicated that while the original measures were deemed acceptable and the physical consequences were not in need of further adaptation, content areas to increase relevance to AI/AN adults included culture, spirituality, family, and community. Participant-relatives also preferred holistic item content that included the importance of all components of health, not focusing on alcohol use alone. Previous research has also highlighted the need for measures and recovery that center on culture and family. Our findings support the trend that after decades of substance use research pathologizing many AI/AN people, communities are now turning to assessments that focus on connectedness (33), spirituality (34), and cultural engagement (35). The development and application of which continues to grow (36).

The addition of cultural items is powerful and can inform future measurement development and interventions. While much non-Indigenous addiction assessment and treatment is individualistic (37), the recommended items emphasized connectedness (38, 39), while acknowledging historical (5), systemic and structural racism (40). Measures and interventions that are limited to individualistic concepts and constructs will miss the richness of holistic conceptions of health, substance-related harms, protective factors, and facilitators of recovery for AI/AN people, and likely other Indigenous and collectivistic populations. Working in partnership with AI/AN client-relatives to uncover the harms of substance misuse has the potential to bolster motivation to change and recovery, at both the individual and social levels, as well as finding ways to repair those harms and build upon cultural strengths.

Our results also underscore the difficulty in defining cultural engagement and appropriate activities that might be endorsed throughout many diverse AI/AN communities. Content validity may be enhanced through direct adaptations to items around cultural engagement. Within our sample, not all the items that described cultural or spiritual activities were seen as universally applicable. For example, not everyone agreed that dancing supports recovery. It was, however, agreed that spirituality would be an important component to include in the measures. Ultimately, operationalizing cultural engagement among AI/AN communities is complex and calls for additional strategies. One strategy might be to identify categories or domains (e.g., physical, spiritual) within a community that is culturally appropriate, instead of specific activities (e.g., powwows). As highlighted elsewhere, another approach might be to emphasize cultural identification or beliefs instead of behaviors (36).

While pushing back on a deficit model, it is also important to identify the reasons for health inequities among Indigenous populations. Historical and intergenerational trauma, colonial/social determinants of health, and policy are major contributors to health inequities (5, 41) but are rarely addressed in substance use-related measures or in interventions. The interviews we completed underscored that measures should include realities such as disproportionate justice involvement and lack of employment opportunities, while simultaneously remaining strength based. Furthermore, including factors related to structural racism reminds us of the ethical obligation to address harms, perhaps most efficiently through policy changes (e.g., increased economic opportunities, reduced alcohol outlets in racially and ethnically diverse, under-resourced neighborhoods).

Staying focused on prevention and strengths, our findings also support increased access to traditional healing and cultural revitalization. Currently, there are 1115 Medicaid waivers approved in Arizona, California, New Mexico, and Oregon. Through these demonstration pilots, the Indian Health Service, Tribal facilities, and urban Indian organizations can provide cultural services as a reimbursable modality for Medicaid beneficiaries (42). Initiatives like these are important to alcohol assessment as they may aid in measurement-based care by connecting individuals to services that could help reduce negative alcohol consequences, with the possibility of reducing AI/AN health inequities on a larger scale. Ultimately, more community-driven clinical assessments that take a systemic and holistic view of healing from colonial-settler harm are needed.

The study should be interpreted by keeping in mind a few strengths and limitations. Though drawn from a national sample, alongside a rural reservation community, our findings may not generalize to other AI/AN communities. Our inclusion criteria were developed from previous research and included self-report, potentially further limiting the generalizability of our research. Participant-relatives were also from culturally diverse communities, which may be viewed as both a strength and a limitation. Future research might consider these factors when developing its inclusion criteria.

Our Scientific Review Panel and other partners did not recommend analyses related to potential sub-group data convergence or divergence. For example, we did not explore whether differences in age across the sample influenced perceptions. In addition, we assessed the overall experiences of participant-relatives and did not conduct sub-group analysis among the primarily urban and female sample. Additional research and analysis into potential similarities or variations between these groups may further illuminate impactful elements in measurement development. Overall, the guidance from a national Scientific Review Panel that included community-based practitioners and those with lived experience increased the internal validity of the research.

### Future directions

Our team has integrated feedback from the qualitative interviews into the finalized DrInC and SIP adapted measures. We will begin piloting a national online version of the two adapted measures with 150 self-identifying AI/AN adults. A subset of 50 adults will be randomly selected to complete the assessment again 1 week later to assess test–retest reliability. Additional measures will also be administered to determine convergent and discriminant validity, including those related to addiction severity, cultural identity, spirituality, discrimination, and mental health. Future research may further explore the meaning of cultural practices (e.g., sweats, round dances) in terms of spiritual renewal, community cohesion, and cultural identity restoration, in addition to the mechanisms underlying the observed effects (e.g., sense of belonging). Further considerations might also include differences from Western recovery models (e.g., individual behavior change).

Findings support the partnership of a national Scientific Review Panel comprised of both Indigenous and non-Indigenous members with expertise to support the identification of areas to culturally center existing alcohol use measures by prioritizing cultural strengths, removing stigmatizing language, and focusing on reasons to be healthy. Interviews underscored the need to emphasize culture, prevention, and recovery supports when addressing alcohol misuse with AI/AN adults. Future research must continue to adapt and co-develop substance use measures with AI/AN people, highlighting strengths and ways to improve whole-person health.

## Data Availability

The datasets presented in this article are not readily available but reasonable request for data can be made to the first author and will be considered by the participating Tribes in accordance with existing data-sharing agreements. Requests to access the datasets should be directed to katherine.hirchak@wsu.edu.
